# Radiomics model using preoperative computed tomography angiography images to differentiate new from old emboli of acute lower limb arterial embolism

**DOI:** 10.1515/med-2023-0671

**Published:** 2023-03-06

**Authors:** Rong Liu, Junlin Yang, Wei Zhang, Xiaobo Li, Dai Shi, Wu Cai, Yue Zhang, Guohua Fan, Chenglong Li, Zhen Jiang

**Affiliations:** Department of Radiology, The Second Affiliated Hospital of Soochow University, Suzhou, China; Department of Radiology, The First Affiliated Hospital of Anhui Medical University, Hefei, China; GE Healthcare Life Science, Shanghai, China; Department of Vascular Surgery, The Second Affiliated Hospital of Soochow University, Suzhou, China

**Keywords:** arterial embolization, X-ray computed tomography, radiomics, thrombosis

## Abstract

Our purpose was to devise a radiomics model using preoperative computed tomography angiography (CTA) images to differentiate new from old emboli of acute lower limb arterial embolism. 57 patients (95 regions of interest; training set: *n* = 57; internal validation set: *n* = 38) with femoral popliteal acute lower limb arterial embolism confirmed by pathology and with preoperative CTA images were retrospectively analyzed. We selected the best prediction model according to the model performance tested by area under the curve (AUC) analysis across 1,000 iterations of prediction from three most common machine learning methods: support vector machine, feed-forward neural network (FNN), and random forest, through several steps of feature selection. Then, the selected best model was also validated in an external validation dataset (*n* = 24). The established radiomics signature had good predictive efficacy. FNN exhibited the best model performance on the training and validation groups: its AUC value was 0.960 (95% CI, 0.899–1). The accuracy of this model was 89.5%, and its sensitivity and specificity were 0.938 and 0.864, respectively. The AUC of external validation dataset was 0.793. Our radiomics model based on preoperative CTA images is valuable. The radiomics approach of preoperative CTA to differentiate new emboli from old is feasible.

## 1 Introduction

Acute limb ischemia (ALI) refers to the abrupt stenosis or occlusion of the lower extremity arteries due to various causes, resulting in insufficient blood supply to the limbs and circulatory disorders. Because ALI progresses rapidly, early screening and timely and correct treatment are vital in saving patients’ lives and limbs. Acute arterial embolization and acute thrombosis are two common causes of ALI. Acute lower extremity arterial embolization (ALEAE) refers to acute ischemic lesions in limbs and tissues caused by blood being pushed to the distal arteries and obstructing arterial blood flow after the heart or near-end artery wall embolus is detached. Emboli originate from the heart, and most of the older ones are left atrial thromboses with reduced systolic capacity and blood retention. The clinical manifestations of the two causes are similar, but the clinical treatments for different causes differ [1]. Because old emboli may be found at the heads of whole emboli in acute arterial embolization, the fundamental problem cannot be solved only by thrombolytic therapy, and the removal of emboli is imperative for patients. However, acute thrombosis is caused by lower extremity atherosclerosis. Luminal stenosis leads to thrombolysis in some patients. The effects are not good because if arterial embolization is mistakenly used to remove emboli, then patients may require repeated stenting. This is a waste of money, time, and trust. In other words, if we can find the old emboli at the head, to some extent, we can select the appropriate treatment.

The diagnosis of both ALE and ALI is mainly based on digital subtraction angiography (DSA). However, DSA has some limitations in practical application [2]. First, DSA is an invasive examination. Second, it is closely related to the operator’s experience. If the operator cannot complete the diagnosis after a single contrast injection, the amount of contrast agent and radiation the patient receives increase dramatically.

In clinical practice, noninvasive imaging modalities play an important role in the diagnosis of ALI [3]. However, the age of thrombosis is still a dilemma for medical imaging specialists. Although, venous duplex ultrasonography, combining color flow Doppler imaging with compression ultrasonography, which is the current gold standard first-line investigation for imaging deep vein thrombosis, is unable to differentiate between acute and chronic thrombi [4]. Some studies have also used magnetic resonance imaging for lower extremity thrombosis in older adults, but its clinical application has been greatly reduced by its long scanning time, insensitivity to calcification, and metal limitations. Computed tomography angiography (CTA) is a kind of examination of acute lower extremity arterial ischemia [5]. It is noninvasive, rapid, and also shows other abdominal and pelvic lesions [6]. Preoperative lower extremity CTA can help to determine the best surgical approach and devise the best surgical plan, so it is unfortunate that it cannot perform thrombosis aging more accurately.

Recently, the emerging field of radiomics has expanded its use and improved the understanding of medical imaging. Radiomics is the process of extracting quantitative features from radiological images via high-throughput analysis and selecting features to build a signature for a complete tumor characterization [7,8,9].

The core task of radiomics is to automatically analyze image feature data to obtain valuable information for disease diagnosis, prognosis, and prediction. The process of radiomics can be divided into several parts: acquiring high-quality, standardized images, and determining tumor regions using a segmentation algorithm or clinical expert sketching; extracting large numbers of image features from the tumor region and selecting the most effective features; and establishing a prediction model by analyzing the selected features and results through machine learning. The research methods of radiomics can obtain useful information from images and have great potential to improve the value of diagnosis and prognosis.

To date, few studies have applied radiomics to the prediction of lower extremity arterial thrombosis. Therefore, the purpose of this study was to differentiate new from old emboli of acute lower limb arterial embolism of femoral popliteal artery by preoperative CTA-based radiomics model.

## 2 Materials and methods

**Figure 1:**
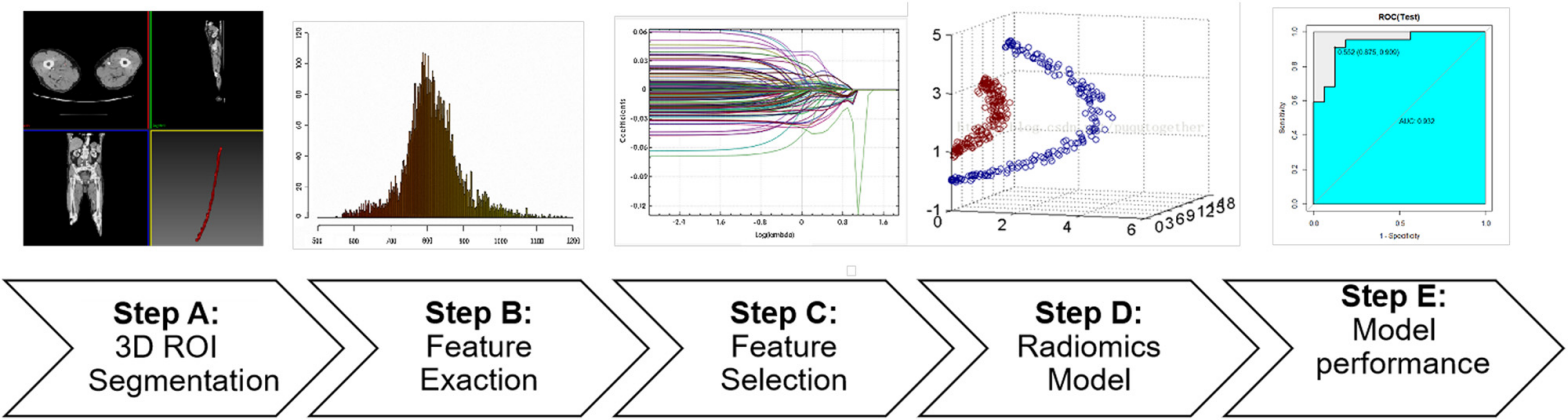
Research workflow. A: Three-dimensional (3D) ROIs were segmented from CTA images; B and C: Quantitative imaging texture features were extracted and selected to construct the radiomics model; D: Three different radiomics models were constructed; E: ROC curve analysis was used to evaluate the performance of the model.

The workflow of this research is presented in Figure 1.

### 2.1 Patients population

This retrospective study was approved by the ethical review board of our hospital (IRB number: JD-HG-2021-32). Informed consent was obtained from all patients. Fifty-seven patients with acute arterial embolism in our hospital were enrolled in the study between January 2015 and December 2017, including 45 male and 12 female adults aged from 40 to 88 years, with an average age of 73.52 years. In total, 95 ROIs were detected in these patients, including old emboli (50) and new thromboses (45).

**The inclusion criteria were:**

a) Clinical manifestations of acute onset, manifested by varying degrees of sudden limb pain, followed by numbness, paleness, cyanosis, dyskinesia, cold limbs, arterial pulse weakening, or disappearance of symptoms;
b) Gross specimen showing that the thrombosis head was sclerotic and the tail thrombosis was soft; pathological diagnosis showing that the head thromboses was white and the tail thromboses was red;
c) Embolus taken immediately after CTA examination of the lower extremities at DSA.

**The exclusion criteria included:**

a) Artifacts in images;
b) Unclear popliteal artery lumen;
c) Images could not be matched;
d) Nephrotic syndrome.

**Figure 2:**
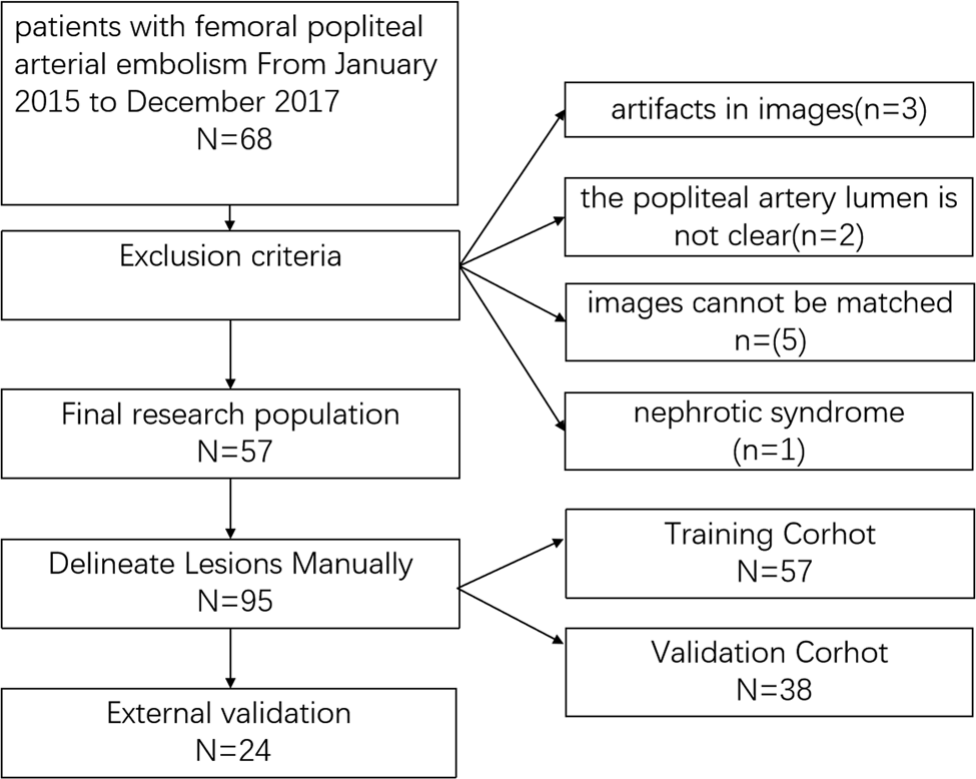
Flow diagram of patient selection.

Figure 2 shows the details of patient selection.

### 2.2 CT imaging

Before therapy, all patients received conventional CT scans using the GE 64-layer LightSpeed VCT scanner. The scan ranged from the lower abdominal aorta to the sole of the foot. The patient assumed the advanced scanning position of the supine foot, and the foot was fixed to the inversion. The scan conditions were tube voltage 120 kV, tube current 260 mA, and pitch 0.8. The contrast agent was 370 mgI/mL, which was injected into the cubital vein at 4 mL/s, and the amount was 100 mL. After the injection, it was diluted with 30 mL of normal saline. The scan was performed using the contrast agent to track the prep smart technique, and a region of interest (ROI) was placed at the level of the abdominal aorta. The field value was 150 HU, and the delay was 10 s after the trigger. Blood vessel volume data were transmitted from CT scans to a GE ADW4.6 stand-alone workstation for volume rendering, maximum intensity projection, curved planar reconstruction, multiplanar reconstruction, and multi planar reformation. CT scan images were exported in DICOM format.

### 2.3 Fogarty balloon catheter thrombectomy

The anesthesia method was 2% lidocaine local anesthesia, and longitudinal incision of the upper part of the femoral artery was performed. After the artery was exposed, systemic intravenous heparinization was achieved with 5,000 units of heparin, and a longitudinal incision was made in the anterior wall of the artery. After the catheter was inserted into the blood vessel, the balloon was dilated. It was then slowly withdrawn, removing the emboli and secondary thromboses. A sign of successful thrombectomy is a violent jet of blood near the heart with turbulent blood flow to the distal end. Angiography indicates smooth blood flow with no filling defect.

### 2.4 ROI and texture extraction

**Figure 3:**
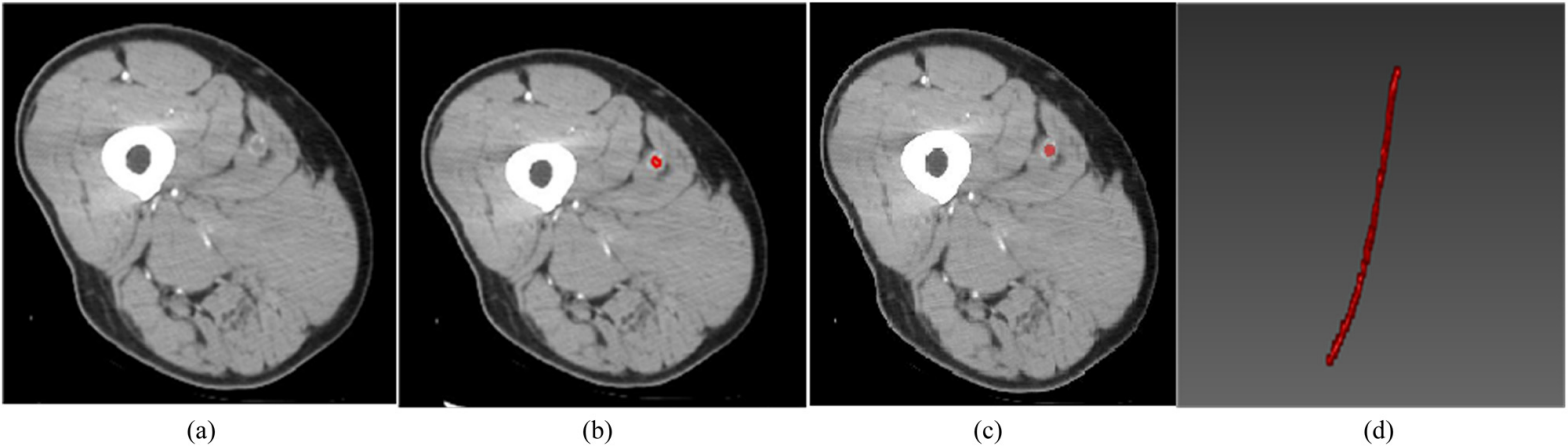
Example of 3D ROI segmentation. Male patient aged 65 years with thrombosis in the right lower extremity deep vein. (a) CTA imaging. (b and c) Image segmentation performed on CTA images. (d) 3D ROI segmentation.

Lesions were outlined as ROIs, and feature extraction was performed using the Artificial Intelligence Kit software package (version 3.1.0, GE Healthcare (Shanghai, China) Co., Ltd). All data are sketched on the CTA axis map. The thrombosis lesions corresponding to the head and tail of the general specimen are selected, and the ROI is placed along the contour of the lesion. The calcified blood vessel region of the pixel threshold is elucidated to further adjust the ROI, and then the respective image ensemble feature values within the ROI are extracted. ROI outlining of the thrombosis was performed by an experienced imaging specialist and examined by another imaging specialist. Then, ROIs from multiple levels of the same lesion were merged into a three-dimensional (3D) ROI. Figure 3 shows an example of the delineated 3D ROI. Radiomics features were extracted from the axial CTA images. The computer-derived features, including first-order statistics, gradient-based histogram features, second-order Haralick textures, and form factor parameters, were calculated based on the voxels in the delineated ROI. The extracted radiomics features include: (1) gray histogram: kurtosis, skewness, mean, variance, and percentile (10%, 25%, 50%, 75%, 90%); (2) Gray level co-occurrence matrix: contrast, angular second order, correlation, and entropy; (3) gray run length matrix: long run enhancement, short run enhancement, fractional run, and run length non-uniformity measure; (4) form factor parameters: sphericity, surface area, compactness, and volume; (5) Haralick features: Haralick correlation, angular second moment, contrast, Haralick entropy, variance, sum average, sum entropy, and inverse difference moment; (6) gray level size zone matrix: small and large zone emphasis, gray-level nonuniformity, zone percentage, and gray-level variance.

### 2.5 Feature selection and radiomics model

Despite the fact that 396 features were exacted through texture analysis, not all of them would be useful for differential diagnosis. We adopted a series of dimensionality reduction methods to avoid overfitting and feature selection methods to identify the optimal features to include in the radiomics model. ANOVAs and Mann–Whitney *U* tests were first used to delete less useful features, and the least absolute shrinkage and selection operator (LASSO) method was also performed to explore which features correlated best with histopathology. The LASSO method is a kind of compressed estimation that refines the model by constructing a penalty function, which first determined the hyper-parameter λ using 10-fold cross validation, and the optimized λ corresponding to the minimized binomial deviation was selected and used to compress some coefficients and set some coefficients to zero. Finally, 34 of the most useful predictive biomarkers with non-zero coefficients were investigated to construct the radiomics prediction model.

To distinguish the difference between new and old thromboses, the 95 ROIs from 57 patients were randomly divided into two groups: 60% of the ROIs were assigned to the training set (*n* = 57), and the rest were assigned to the internal validation set (*n* = 38). The training set was used to establish the predictive radiomics model, and the internal validation set was used to evaluate the model’s performance. Besides, the external validation dataset was also prepared to evaluate the model’s performance to validate the model’s generalization.

For model construction, we used three of the most common machine learning methods available and selected the best prediction model according to performance through iterative verification.

Support vector machine (SVM) is a common discrimination method in machine learning. It is a supervised learning method that is commonly used for pattern recognition, classification, and regression analysis. The SVM method maps the sample space into a high-dimensional feature space that can even have infinite dimensions (Hilbert space) through a nonlinear mapping (*p*), transforming the problem of nonlinear separability in the original sample space into the feature space. In our SVM model, we used the radial basis function (RBF) to convert the data so that the original could not be separated into linear functions. RBF is a real-valued function that only depends on the distance from a specific point. In SVM, the coefficient *C* is the cost function’s penalty coefficient, and higher values are associated with less error tolerance. To increase accuracy, we set the coefficient *C* to 1,000.

In recent years, deep learning has been widely employed in various fields, especially for automatic lesion segmentation. Deep learning is a series of new structures and methods that can be evolved by multi-layer ANNs with additional layers. The back-propagation (BP) algorithm is the most important part of feedforward neural networks (FNN). It is a supervised learning algorithm that is often used to train multilayer perception networks, and it is defined by the activation function used by each artificial neuron (i.e., node) being micro. Multilayer FNNs have one input layer, one or more hidden layers in the middle, and one output layer. In our model, we used the BP method with ten layers, with each layer being equivalent to a single-layer FNN. This network construction forms dimensional hyperplanes, which helps to achieve more complex classification of input patterns.

Random forest (RF) is a new, highly flexible machine learning algorithm that has a wide range of applications. It integrates multiple basic decision tree units by ensemble learning, and it is an ensemble learning method.

Intuitively, each decision tree is a classifier (assuming the classification problem is now addressed). Thus, for an input sample, *N* trees will have *N* classification results. RF integrates all of the classification voting results and assigns the most voted category as the final output. This article used 500 trees, and the number of variables attempted at each split was 5. We created an RF model because it has state-of-the-art accuracy among current algorithms and gives estimates of which variables are important in the classification.

## 3 Statistical analysis

All statistical analyses were conducted using R (version 3.4.7, http://www.Rproject.org). Two-tailed testing was used in all statistical analyses, with the level of statistical significance determined as *p* < 0.05. Before model construction, two-step preprocessing was performed on the data obtained from texture analysis: first, the missing data were replaced with medians to eliminate their impact on the final results. Second, to reduce the effects of dataset size on the results, standardization was performed.

To test the radiomics model’s performance, receiver operating characteristic (ROC) curves were used, and the area under curve (AUC) with 95% confidence interval (CI) was analyzed. Each model was iterated 1,000 times, and the model with the best AUC was selected. The DeLong’s test was used to determine the significance of AUC differences among the three classifiers.

## 4 Results

### 4.1 General information

Two site datasets were included in the study, one site contained 57 patients, 95 total ROIs were delineated: 45 were new arterial thromboses (47%), and 50 were old arterial emboli (53%). The second site consisted of 21 patients, 24 ROIs were delineated: 14 were new arterial thromboses (58%), and 10 were old arterial emboli (42%).

### 4.2 Feature selection

**Figure 4:**
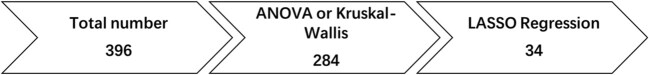
Feature extraction.

Figure 4 shows the two feature selection steps and the number of remaining features in each step.

The features after feature exaction are listed in Table 1.

**Table 1:** Radiomics: predictive factors

Feature type	Feature name
Gradient-based histogram features	Percentile (10%, 15%)
	Relative deviation
Texture parameters	Correlation (offset 4, 7)
	Cluster shade
	Cluster prominence
GLCM	GLCM energy (angles 0°, 90°, 135°)
	GLCM entropy (angles 0°, 90°)
Inverse difference moment	Inverse difference moment (offset 1, 7)
RLM	Run length nonuniformity (all directions, offset 1, 4, 7)
	Short run emphasis
	Long run emphasis
	Long run high-grey level emphasis
	Low grey-level run emphasis
Form factor parameters	Surface area
	Compactness (1, 2)
	Volume CC
	Volume MM
	Sphericity
	Spherical disproportion

Through feature selection, we found six different types of image features that can influence the model construction: gradient-based histogram features (3), texture parameters (4), GLCM (5), Inverse difference moment (2), RLM (13), and form factor parameters (7).

### 4.3 Model performance

**Figure 5:**
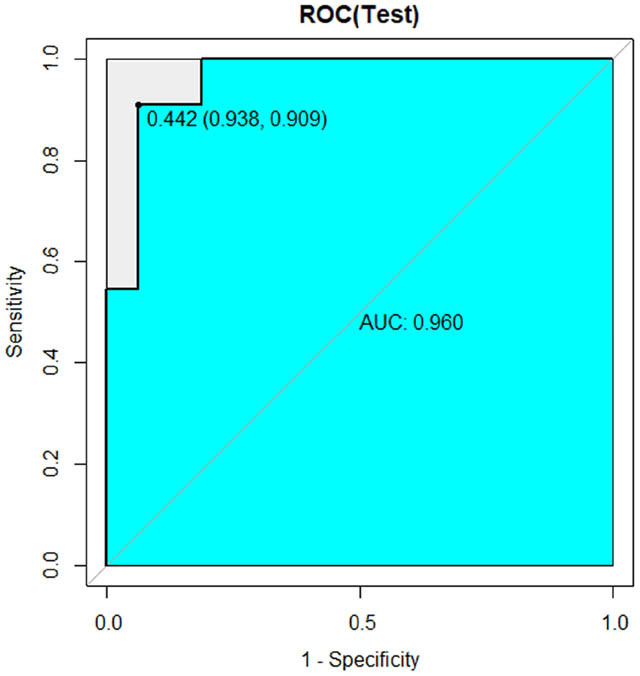
ROC curve of BP model in internal validation dataset.

**Figure 6:**
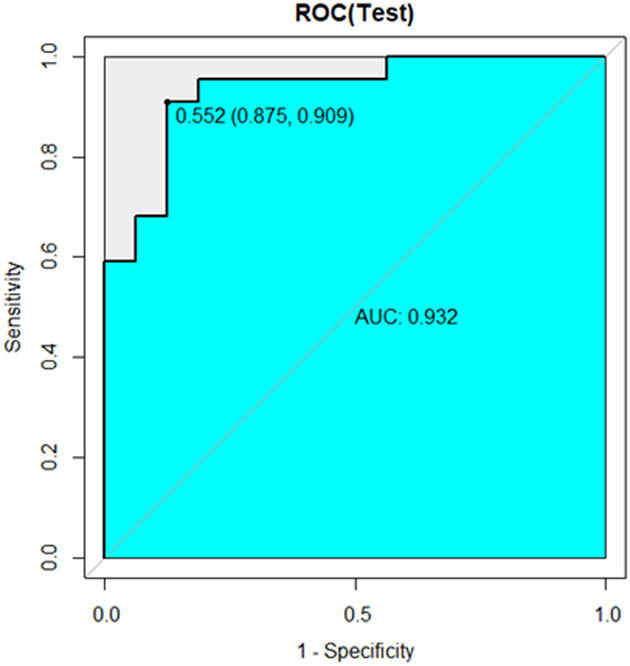
ROC curve of RF model.

**Figure 7:**
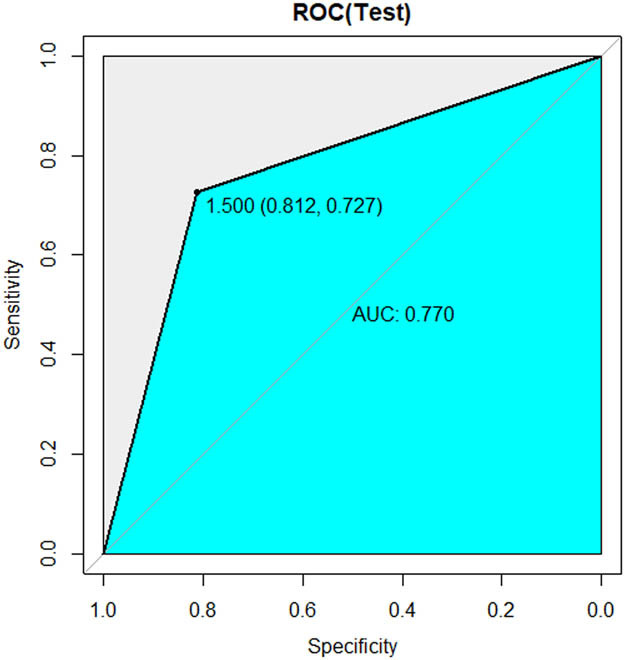
ROC curve of SVM model.

**Figure 8:**
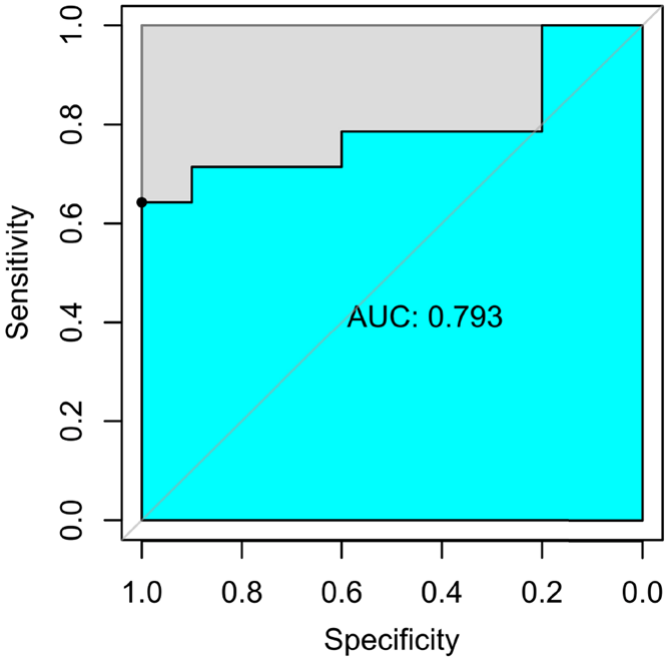
ROC curve of BP model in the external validation dataset.

Table 2 shows the sample distribution of the training and internal validation datasets. As shown in Table 3 and Figures 5–7, all three models had high performance to distinguish the new emboli from old, but the BP model was the best one, in which the AUC value and 95% CI were 0.960 and (0.899–1), respectively. Compared to the other two models, *p* values of the DeLong’s test were both less than 0.05. The BP model was also validated in an external validation dataset, and the AUC was 0.793 (95%CI: 0.601–0.985) (Figure 8).

**Table 2:** ROI distribution

	Total	New thrombus	Old thrombus
Data	95	45	50
Training set	57	27	30
Validation set	38	18	20
External validation	24	14	10

**Table 3:** Comparison between SVM, BP, and RF

	SVM model	RF model	BP model	
Validation	Internal	Internal	Internal	External
AUC	0.770	0.932	0.960	0.793
95% CI	0.633–0.907	0.853–1.000	0.899–1	0.601–0.985
Accuracy	0.763	0.842	0.895	0.792
Sensitivity	0.813	0.875	0.938	0.643
Specificity	0.727	0.812	0.864	1.000

## 5 Discussion

To the best of our knowledge, this is the first study that used preoperative CTA to differentiate new emboli from old. ALI is approximately five times more prevalent in the lower extremities than the upper extremities [10], so we studied only patients with femoral popliteal artery embolism. Perioperative morbidity and mortality after ALI are also high, ranging from 20–40% [11]. The sudden interruption of the blood supply to the lower extremity arteries and insufficient time to establish collateral blood circulation may lead to serious blood supply insufficiency. Ischemic symptoms occur more quickly, and the degree of ischemia is more serious. Thus, the importance of early diagnosis and clinical treatment are obvious. The age of patients in this study was in the range of 40–88 years, and most of them were older adults. Based on our experience, DSA thrombectomy was more suitable for this kind of patients than traditional surgical thrombectomy after definite diagnosis of CTA before operation. In addition, for some patients with both arterial thromboses and lower extremity atherosclerosis, or patients with a long duration of arterial thrombectomy or thrombolysis is less effective, and balloon dilatation and stent treatment are required. Thus, preoperative CTA to distinguish new emboli from old can provide useful guidance for treatment.

Previous research reported used duplex scanning to differentiate embolic from thrombotic acute arterial occlusion [12]. In their study, the clinical data were not sufficient to differentiate the embolic from thrombotic occlusion. The acuteness of presentation was not clinically or statistically different between embolic and thrombotic groups. The site of occlusion, state of the arterial wall (healthy or affected by atherosclerosis), and the presence of calcification or collaterals were not sufficient to differentiate embolic from thrombotic occlusion. Just the arterial diameters at the site of occlusion were useful. Thus, we hypothesized whether radiomics could be used to differentiate. Radiomics methods have been widely spread because they can transform medical images into high-dimensional quantitative textural features and use the optimal features to guide clinical decision making [13]. Most previous radiomics studies focused on tumors. Compared with simple subjective qualitative features based on tumor lesions, quantitative image features can describe tumor heterogeneity more comprehensively and quantitatively. Such quantitative features have been used in many medical imaging applications to assist with disease diagnosis and treatment, and they can compensate for the shortcomings of traditional qualitative diagnosis [14,15,16,17,18]. The results show that the various imaging features extracted from CT images can reflect the tumor’s potential biological characteristics and heterogeneity, which can be used to predict the postoperative prognosis and evaluate curative effects on patients [21,22]. Recently, several studies reported that the value on atherosclerosis and thrombus radiomics features derived from NCCT and CTA are more predictive of recanalization [23,24].

In this study, we devised a radiomics method to distinguish new from old emboli of acute lower limb arterial embolism. Although radiomics has been widely applied, radiomics methods differ, and their repeatability is poor. Because SVM, RF, and ANN have been recognized as three major methods in supervised machine learning, we used them to construct three radiomics models and chose the one with the best predictions throughout 1,000 iterations. In this study, we extracted 34 predictive factors based on angiographic features of preoperative CT images. We found that the relationships between the uniformity of the gray distribution in the ROI, the degree of texture of grooves, and three-dimensional shape are the main discriminant factors of lower limb acute arterial embolism. The FNN radiomics prediction model had the largest area under the ROC curve: 0.960 (0.899–1). When the models were trained with the validation dataset, they retained high performance in terms of accuracy, sensitivity, specificity, and AUC for achieving the correct diagnosis. Thus, the results indicate that the radiomics approach of using preoperative CTA images to differentiate new emboli from old is feasible and can effectively help with clinical preoperative diagnosis and evaluation.

## 6 Limitation

First, it was a single-center retrospective study with potential bias; second, texture features were extracted from manually segmented data, and it was difficult to exclude small blood vessels within the thromboses, which may affect the accuracy of some features; third, all patients with arterial embolism but no arterial thrombosis were selected. In our next study, we will select patients with arterial thrombosis for validation.

## 7 Conclusion

In conclusion, despite the fact that CT-based radiomics methods cannot be expected to replace pathological diagnosis, as a noninvasive approach, the radiomics prediction model based on angiographic labeling of preoperative CTA images is valuable for distinguishing the new femoral emboli from old. This noninvasive method can provide individualized preoperative predictors for clinicians and patients. In addition, the method performs radiomics analysis on existing images without additional cost and thus has the potential for extensive clinical use.
